# Saccular functions differ for Meniere’s disease with and without coexisting headaches

**DOI:** 10.3389/fneur.2023.1141388

**Published:** 2023-04-14

**Authors:** Takaki Inui, Tatsuro Kuriyama, Kou Moriyama, Takeo Shirai, Tomoyuki Sudo, Yusuke Ayani, Akiko Ozaki, Yuko Inaka, Michitoshi Araki, Shin-Ichi Haginomori, Ryo Kawata

**Affiliations:** Department of Otorhinolaryngology – Head and Neck Surgery, Osaka Medical and Pharmaceutical University, Takatsuki, Japan

**Keywords:** Meniere’s disease, coexisting headaches, migraine, gender-based difference, saccular function

## Abstract

**Objectives:**

To elucidate the differences between the cases of Meniere’s disease (MD) with and without coexisting headaches, especially migraine. The clinical characteristics and vestibular functions are compared.

**Subjects:**

Fifteen patients with definite unilateral MD without headaches (MD/H−; 10 males and 5 females; mean age of 55.8 years), and 20 patients with definite unilateral MD with headaches (MD/H+; 3 males and 17 females; mean age of 54.4 years; 15 cases of migraine without aura and 5 cases of suspected migraine or tension-type headache) were enrolled.

**Methods:**

The medical records, caloric test results, and cervical vestibular evoked myogenic potential (cVEMP) of the patients were reviewed. A monothermal caloric test by injection of cold water was performed, and canal paresis was assessed. cVEMP was recorded using 500 Hz short tone bursts, and the asymmetry ratio using the corrected amplitude of p13–n23 was determined.

**Results:**

The patients in the MD/H− group were predominantly male, whereas more female patients were seen in MD/H+ group (*p* = 0.004). In the MD/H+ group, the frequency of vertigo and the dizziness handicap inventory (DHI) values were significantly higher than those in the MD/H− group (*p* = 0.045, <0.001, respectively). There was no statistical difference in the ages, duration of illness, or the hearing levels between both groups. The caloric testing results were abnormal for 10 of the 13 MD/H− cases, and 14 of the 16 MD/H+ cases, which revealed no significant difference between both groups. The cVEMP results revealed positive saccular dysfunction based on the asymmetry ratio of 4 of the 15 MD/H− cases, and 14 of the 20 MD/H+ cases; it was significantly more prevalent in the MD/H+ group than in the MD/H− group (*p* = 0.018). Multivariate analysis of sex, frequency of vertigo, DHI, and cVEMP results showed significant differences only in the cVEMP results (*p* = 0.049).

**Conclusion:**

The present study revealed differences in patients with MD depending on the presence or absence of headaches. MD without headaches showed a significant male preponderance. MD with coexisting headaches was more associated with severe saccular dysfunctions than MD without headaches. Concomitant headache may affect the manifestations of the vestibular function, especially in the sacculus, in MD cases.

## Introduction

Meniere’s disease (MD) (1) and vestibular migraine (VM) (2) are both characterized by recurrent episodic vertigo. MD and VM often coexist, and the term “VM/MD overlapping syndrome” has been suggested for cases of the coexistence of different types of vertigo associated with VM and MD in the appendix of the International Classifications of Headache Disorders, third edition (ICHD-3) (3). Despite each pathology for MD and VM has been suggested, a common condition may be exist for some cases of MD and VM. However, it is yet to be determined if there are differences in the pathology of MD depending on the presence or absence of concomitant migraines. Toriyama et al. (4) reported that definite VM and migraine with vestibular symptoms (but not meeting the criteria of VM) belonged to the same spectrum of disorders, depending on the evaluation of clinical features related to central sensitization. This report indicates the possible relationships between migraine including VM and vestibular disorders including MD. On the other hand, there are a few published studies which indicate more severe conditions of MD cases with migraine compared to MD without migraine (5, 6). These aspects suggest the possibility that MD may have different manifestations depending on the association with headaches, especially migraine. Researchers have compared semicircular canals and saccular functions of MD and VM: the results of these studies revealed that the caloric test and cervical vestibular evoked myogenic potential (cVEMP) may be useful for diagnostic differentiation of these diseases, whereas some of the conclusions are not consistent (7). Therefore, investigation of the relationship between the presence or absence of headache and MD, by evaluating vestibular examinations such as caloric test and cVEMP, may be helpful to understand the affinity of MD and VM.

The present study aimed to elucidate the differences between the clinical characteristics of MD cases depending on the presence or absence of coexisting headaches, especially migraine.

## Materials and methods

### Participants

In the present study, we analyzed the results of examinations of the peripheral vestibular system in patients with recurrent vertigo. The medical records of 35 patients, including 15 with definite unilateral MD without headache (MD/H− group), and 20 with definite unilateral MD with headaches (MD/H+ group), who visited our institute between October 2018 and November 2022 were reviewed retrospectively. In the MD/H+ group, 15 patients had migraine without aura (MOA) and 5 were suspected of MOA or tension-type headaches. The patients were diagnosed with MD based on the criteria of the Bárány Society (1), and headaches based on ICHD-3 (3). Patients were asked whether they had concomitant headaches at the end of the medical interview during their first visit if they had not complained of headaches. Association between headaches and vertigo attacks were assessed by using the self-reported dizziness and headache diary (8). The patients who had more than half of their headaches associated with vertigo attacks were excluded from this study because they can be diagnosed as having VM or VM/MD overlapping syndrome (as a result, 25 VM and 5 VM/MD overlapping syndrome cases were excluded during the study period). The frequency of vertigo attacks per month for the previous 6 months and the duration of illness were confirmed *via* medical interviews. The vestibular examinations were guided by the results of the caloric and cervical vestibular myogenic potential (cVEMP) tests. These examinations were performed during the interictal phase within 3 weeks of the first visits of the patients.

Informed consent was obtained from all the participants. This study was performed according to our clinical practice guidelines and all relevant tenets of the Declaration of Helsinki. This study was approved by the ethics committee of our institute (approval number 2426-1).

### Dizziness handicap inventory

Patients were administered the Japanese version of the dizziness handicap inventory (DHI) (9) at the first visit to quantitatively assess the self-perceived impairment in daily life.

### Hearing level

Audiometric testing data were obtained on the day of first visit and the pure-tone averages of threshold values at 0.5, 1, 2, and 3 kHz were calculated. We averaged the values of 2 and 4 kHz because we did not routinely determine the 3 kHz values; this is a modification of the guideline of the American Academy of Otolaryngology and Head and Neck Surgery published in 1995 (10, 11).

### Caloric test

Canal paresis (CP) of the lateral semicircular canal was evaluated using a monothermal caloric test with electronystagmography. Cold water (20°C, 5 mL) was injected into the external auditory canals for 10 s, and the resultant nystagmus was recorded in the dark with the eye open. CP was assessed based on the maximum slow-phase velocity measured by electronystagmography. We classified maximum slow-phase velocities of <20°/s and CP% of >25 as significant unilateral weakness of the responses. The 25% cutoff for the monothermal caloric test is optimal for detecting CP, as evaluated by bithermal caloric testing (12).

### cVEMP

Based on a previous report, cVEMPs were recorded ipsilaterally while the patients were seated upright and their heads were turned to the opposite side using the Eclipse system (Interacoustics, Middelfart, Denmark) (13). Monoaural 500 Hz short-tone burst stimuli were applied *via* intra-auricular speakers with foam ear tips. We analyzed the first biphasic response (p13–n23) of the averaged responses to 50–100 stimuli maintained by each tonic electromyogram. The asymmetry ratio (AR) was calculated using the corrected amplitude (CA) of p13–n23 as follows:


AR=100×(CAu−CAa)/(CAu+CAa).

Where CAu is the normalized amplitude (p13–n23) of the unaffected side, CAa the normalized amplitude of the affected side, in response to the 500 Hz short-tone burst stimuli. The mean AR was 9.29 (SD, 7.8) for the healthy participants in our institute; therefore, the upper normal limit was considered to be 24.89 (mean + 2 SD).

### Statistical analysis

All statistical analyses were performed using JMP 16^®^ (SAS Institute Inc., Cary, NC, United States). The ages, DHI, and hearing levels of the groups were compared using an unpaired-*t*-test. Frequency of vertigo, duration of illness, and the distribution of the AR values of cVEMP were compared using a Mann–Whitney *U*-test. The sex, prevalences of CP based on the caloric test and positive AR associated with cVEMP in the groups were compared using Fisher’s exact probability test. For multivariate analysis, logistic regression was applied. The standard logistic regression analysis included male patients for sex, frequency of vertigo, DHI, and cVEMP results (dysfunction or WNL depend on positive AR). For all analyses, *p*-values of <0.05 denoted statistical significance.

## Results

### Participants

The patient backgrounds and the results of examinations that were evaluated by univariate analysis are listed in Table 1. The MD/H− group comprised significantly more males than the MD/H+, in other words, more female patients were seen in MD/H+ group (*p* = 0.004). In the MD/H+ group, the frequency of vertigo and DHI values were significantly higher than those in the MD/H− group (*p* = 0.045, <0.001, respectively). There was no statistical difference in the ages, duration of illness, and the hearing levels between both groups.

**Table 1:** Univariate analysis of factors associated with Meniere’s disease with or without headaches.

	MD/H−	MD/H+	*p*-value
Number of cases	15	20	
Sex (male/female)	10/5	3/17	0.004
Age (mean, SD)	55.8, 9.0	54.4, 12.8	0.721
Frequency of vertigo (per month for the previous 6 months, SD)	2.53, 5.9	4.85, 4.0	0.045
DHI	26.6, 14.2	54.25, 21.7	<0.001
Duration of illness (months, SD)	52.1, 50.3	60.9, 80.5	0.934
Hearing levels (dB, SD)	41.3, 19.9	30.7, 16.9	0.107
Caloric test (CP+/CP−)	10/3	14/2	0.632
cVEMP (dysfunction+/WNL)	4/11	14/6	0.018

MD/H−, Meniere’s disease without headache; MD/H+, Meniere’s disease with headache; DHI, dizziness handicap inventory; CP, canal paresis; cVEMP, cervical vestibular evoked myogenic potential.

### Caloric test

The caloric test results were abnormal for 10 of the 13 MD/H− cases, 14 of the 16 MD/H+ cases; there was no significant difference between both groups. Two patients with MD/H− and 4 with MD/H+ did not undergo the caloric test.

### cVEMP

**Figure 1:**
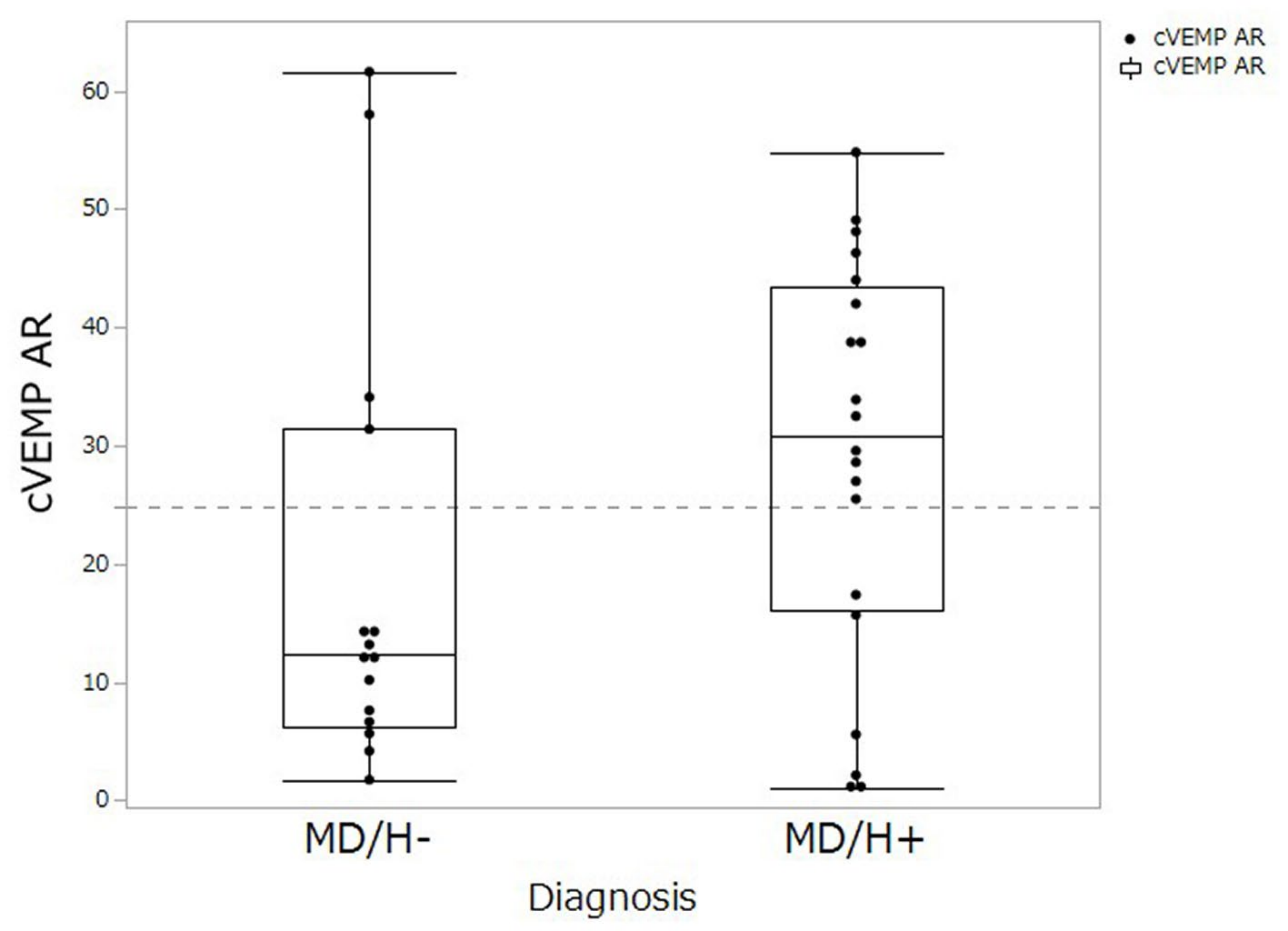
Comparison of cVEMP AR values between MD/H− and MD/H+ groups. The distributions of AR (asymmetry ratio) values on the cVEMP was not differ significantly between the MD/H− group and the MD/H+ group (*p* = 0.122). The dash line shows the upper normal limit (24.89) of AR in our institute.

cVEMP testing revealed saccular dysfunction based on the AR for 4 of the 15 patients with MD/H−, and 14 of the 20 patients with MD/H+. Thus the number of patients with saccular dysfunction, determined using the AR, was significantly higher in the MD/H+ group than that in the MD/H− group (*p* = 0.018). The distribution of AR values was shown in Figure 1.

Following the results of the univariate analysis described above, especially for the factors that revealed significant differences between MD/H− and MD/H+ groups, that is, sex, frequency of vertigo, DHI, and cVEMP results, adjustment of the relevant confounding factors was performed. A subsequent multivariate analysis of these factors showed significant differences only in the cVEMP result (*p* = 0.049) (Table 2).

**Table 2:** Multivariate analysis of factors associated with Meniere’s disease with or without headaches.

	95% confidence interval for partial regression coefficient			*p*-value
Variable	Odds ratio	Lower limit	Upper limit	
Sex	0.061	0.002	1.700	0.100
Frequency of vertigo (per month for the previous 6 months, SD)	1.100	0.776	1.570	0.583
DHI	1.130	0.999	1.280	0.051
cVEMP (dysfunction+/WNL)	43.60	1.020	1870.00	0.049

DHI, dizziness handicap inventory; cVEMP, cervical vestibular evoked myogenic potential.

## Discussion

The findings of the present study suggest different manifestations of patients with MD with or without headaches. The headaches in our MD/H+ cases were predominantly migraines, and the prevalence of vestibular dysfunction, especially related to saccular function, was higher in the MD/H+ group than in the MD/H− groups. In many of the studies on MD have not mentioned the relationships between the vestibular functions and coexisting headaches, to the best of our knowledge.

MD demonstrates a slight female preponderance (14). However, this study revealed a significant female preponderance for MD/H+ and a male preponderance for MD/H−. These gender characteristics are consistent with a previous report (6). The female preponderance for MD/H+ may be a repercussion of the female preponderance for migraine. Our results indicate that MD may be more common in males if they do not have a history of migraine.

The difference between the cVEMP results of the patients with MD/H− and MD/H+ requires the consideration of the relationships between MD and headaches, mostly migraine, particularly for conditions in the inner ear. Many factors exhibited significant differences in the univariate analysis between the two groups, that is, sex, frequency of vertigo, DHI, and cVEMP results, multivariate analysis revealed significant differences only in the cVEMP results. This indicates that the female preponderance in MD/H+ group did not influence the differences in the cVEMP results in the present study. Contrary to this evaluation of the approach dividing the examination results as normal or abnormal by setting a threshold, the AR distribution did not differ significantly between the two groups (Figure 1, *p* = 0.122). However, many cases of MD/H− showed smaller AR values, and two outliers whose AR values might have been elevated because they had received cVEMP testing relatively earlier than the other cases even though their vertigo attacks already subsided (the numbers of days after the most recent vertigo attacks and the AR values were 3 days/AR 58.0 and 4 days/AR 61.6, respectively). Because of the small sample size of the present study, further investigation with larger sample is needed. Experimental studies on migraine have demonstrated that the activation of the trigeminovascular system with the release of vasoactive neuropeptides, such as substance P (SP), calcitonin gene-related peptide (CGRP), and neurokinin A, causes vasodilation of the dura with the release of pro-inflammatory factors and the resultant neurogenic inflammation (15). Furthermore, the presence of SP and CGRP in trigeminal sensory fibers innervating the inner ear and vestibular nuclei has been reported (16, 17). The pathophysiology of VM is proposed that the inner ear plasma extravasation and the release of inflammatory mediators subsequent to the neurogenic inflammation triggered by the activation of the trigeminal-vestibulocochlear reflex, which are related to hypoperfusion of the inner ear affecting the otolith organs, as well as ischemic lesions of the descending otolith pathways in the brainstem (18). Several researchers have reported reduced or absent VEMP responses in patients with VM, which indicate the dysfunction of the vestibular-collic reflex (7, 19). Therefore, in MD/H+ group, hypoperfusion may occur in the inner ear or otolith pathways in the brainstem as like VM additionally on endolymphatic hydrops, resulting in lower cVEMP test scores than those of the MD/H− group. Conditions involving an overlap of the pathophysiologies of MD and migraine, such as disorders affecting aquaporin 4, may be also related to worse saccular dysfunction (20).

On the other hand, the caloric test results of the MD/H− and MD/H+ groups were not different. It is still difficult to explain why only the cVEMP results of the MD/H− and MD/H+ groups were different; the incidence of endolymphatic hydrops in vestibular organs may be a reason. Okuno and Sando (21) reported that severe endolymphatic hydrops were observed most frequently in the saccule of the inner ear in a pathological investigation of the human temporal bone of patients with MD. Therefore, saccule dysfunction may initially be close to borderline before it is affected by migrainous vasospasm, which leads to hypoperfusion of the inner ear.

Most of the studies on MD have not reported the relationships between the results of vestibular examinations and coexisting headaches, to the best of our knowledge. From the recent literature about differentiation between MD and VM by cVEMP testing, evaluation on the usefulness of cVEMP AR is not consistent; Salviz et al. (22) and Dlugaiczyk et al. (23) reported that the AR of cVEMP was significantly higher for MD than for VM, whereas Eliezer et al. (24) reported that there was no significant differences between MD and VM in the results of cVEMP including AR. This discrepancy in results may be related to the different sex demographics of the study cohorts, or the fact that the subjects were not divided by the presence of headaches. Further research on MD with and without headaches and VM may provide new insights in the future. However, it is not be typical to detect headaches from patients with vertigo precisely in the otolaryngology field; therefore, the presence of coexisting headaches in some patients should be missed. Inui et al. (25) highlighted the importance of asking patients with recurrent vertigo if they also suffer from headaches. The study showed that fewer patients complained of concomitant headaches without medical prompts, especially if they had been diagnosed with other vestibular disorders in the past or had different onset times for their vertigo and headaches. It is important to confirm the existence of headaches in patients with vertigo through medical interviews or questionnaires for extended evaluation of MD.

The present study has some limitations. First, headaches in all MD/H+ patients were not be diagnosed definitively as migraine; however, we enrolled such MD patients in this study. Migraine headaches are sometimes difficult to diagnose, especially when they are longstanding, because their typical characteristics have not been established. That is why we enrolled patients likely to have MD with concomitant migraines after confirming that they do not fulfill the diagnostic criteria for other headaches. Additionally, we only used the caloric test and cVEMP in the present study. oVEMP, video head-impulse test, and evaluation of endolymphatic hydrops will contribute to further perception. Comparison with VM and larger samples will allow further investigation of subgroups based on more detailed data, such as the type of headaches, in the future.

## Conclusion

The present study revealed some differences in patients with MD depending on the presence or absence of headaches. MD without headaches showed a significant male preponderance. MD with coexisting headaches was comprised of larger number of female patients and associated with more severe saccular dysfunctions than MD without headaches. Concomitant headache may affect the manifestations of the vestibular function, especially in the sacculus, in MD cases.

## Data availability statement

The raw data supporting the conclusions of this article will be made available by the authors, without undue reservation.

## Ethics statement

The studies involving human participants were reviewed and approved by The Institutional Ethical Review Board of Osaka Medical and Pharmaceutical University (approval number 2426-1). Written informed consent from the participants’ legal guardian/next of kin was not required to participate in this study in accordance with the national legislation and the institutional requirements.

## Author contributions

TI designed the study and wrote the manuscript and performed the statistical analysis. TI, TK, KM, TSh, TSu, YA, AO, and YI collected the data. TI, MA, S-iH, and RK interpreted the data. S-iH reviewed and edited the manuscript. All authors contributed to the article and approved the submitted version.

## Funding

This work was partially supported by the Japan Society for the Promotion of Science Grant (JSPS KAKENHI Grant #22K09693).

## Conflict of interest

The authors declare that the research was conducted in the absence of any commercial or financial relationships that could be construed as a potential conflict of interest.

## Publisher’s note

All claims expressed in this article are solely those of the authors and do not necessarily represent those of their affiliated organizations, or those of the publisher, the editors and the reviewers. Any product that may be evaluated in this article, or claim that may be made by its manufacturer, is not guaranteed or endorsed by the publisher.
